# Wireless Remote Weather Monitoring System Based on MEMS Technologies

**DOI:** 10.3390/s110302715

**Published:** 2011-03-01

**Authors:** Rong-Hua Ma, Yu-Hsiang Wang, Chia-Yen Lee

**Affiliations:** 1 Department of Mechanical Engineering, Chinese Military Academy, Kaohsiung 830, Taiwan; E-Mail: rh.ma@msa.hinet.net; 2 Department of Mechanical and Automation Engineering, Da-Yeh University, Changhua 515, Taiwan; E-Mail: i_am_alt@yahoo.com.tw; 3 Department of Materials Engineering, National Pingtung University of Science and Technology, Pingtung 912, Taiwan

**Keywords:** MEMS, WSN, weather monitoring system

## Abstract

This study proposes a wireless remote weather monitoring system based on Micro-Electro-Mechanical Systems (MEMS) and wireless sensor network (WSN) technologies comprising sensors for the measurement of temperature, humidity, pressure, wind speed and direction, integrated on a single chip. The sensing signals are transmitted between the Octopus II-A sensor nodes using WSN technology, following amplification and analog/digital conversion (ADC). Experimental results show that the resistance of the micro temperature sensor increases linearly with input temperature, with an average TCR (temperature coefficient of resistance) value of 8.2 × 10^−4^ (°C^−1^). The resistance of the pressure sensor also increases linearly with air pressure, with an average sensitivity value of 3.5 × 10^−2^ (Ω/kPa). The sensitivity to humidity increases with ambient temperature due to the effect of temperature on the dielectric constant, which was determined to be 16.9, 21.4, 27.0, and 38.2 (pF/%RH) at 27 °C, 30 °C, 40 °C, and 50 °C, respectively. The velocity of airflow is obtained by summing the variations in resistor response as airflow passed over the sensors providing sensitivity of 4.2 × 10^−2^, 9.2 × 10^−2^, 9.7 × 10^−2^ (Ω/ms^−1^) with power consumption by the heating resistor of 0.2, 0.3, and 0.5 W, respectively. The passage of air across the surface of the flow sensors prompts variations in temperature among each of the sensing resistors. Evaluating these variations in resistance caused by the temperature change enables the measurement of wind direction.

## 1. Introduction

In recent years, emerging Micro-Electro-Mechanical Systems (MEMS) technology and micromachining techniques have been a popular approach to the miniaturization of sensors. More importantly, the functionality and reliability of these micro-sensors has been increased considerably by integrating them with mature logic IC technology or other sensors.

To effectively gauge the weather, it is essential to gather data, such as temperature, humidity, air pressure, airflow direction and velocity over a wide area. Previous studies have reported on the use of MEMS sensors for monitoring individual weather parameters, such as pressure [1], flow rate [2,3], humidity [4,5], temperature, and multi-parameters (two or more) [6–10].

Among the weather parameters, temperature in particular has been recognized as a key factor in the accuracy of weather predictions. Lee *et al.* [11] described the use of Pt resistors as temperature sensors in MEMS-based temperature control systems. Lee and Lee [12] also proposed micromachine-based humidity sensors, with integrated temperature sensors, for signal drift compensation. This study developed a MEMS-based device using thin-film platinum resistors as temperature sensing elements and a nitride-silicon microstructure suspended at a short distance above the surface of a glass substrate (with a stationary electrode) as the movable electrode of a capacitor. The cantilever was coated with a vapor absorbent polymer film (polyimide), in which fluctuations in humidity caused moisture-dependent bending of the micro-cantilever, thereby changing the measured capacitance between the microstructure and the substrate. To compensate for temperature drift in the capacitance signals, the humidity sensor was integrated with a micro resistance-type temperature detector comprising platinum resistors.

In 2008, Lee *et al.* [13] presented a gas flow sensor comprising sensing units to detect the rate and direction of airflow. The airflow rate sensing unit was formed using a micro heater and a sensing resistor produced over a membrane that was released using a back-etching process. The sensing unit for the direction of airflow comprised four cantilever beams set perpendicular to one another and integrated with a piezoresistive structure on each micro-cantilever. Because the cantilever beams were formed after etching the silicon wafer, it was bent upward slightly due to the released residual stress induced in the previous fabrication process. As air flowed through the sensor, the temperature of the sensing resistor decreased and changes in local temperature were determined as the rate of airflow. On the proposed sensor, the direction of airflow was also determined by comparing variations in resistance produced by the deformation of cantilever beams in different directions.

**Figure 1.**
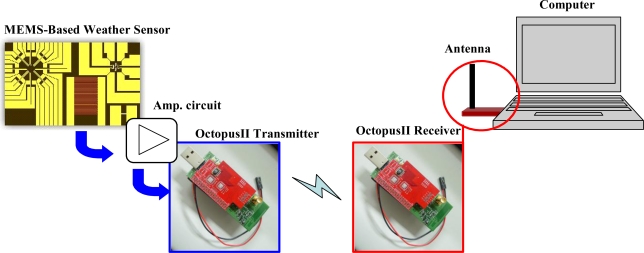
Overview of a wireless remote weather monitoring system.

The current study developed a fabrication process integrating Pt resistor temperature detectors (RTD), Au inter-digitated electrodes (IDEs) and polyimide layers for sensing humidity, Pt-piezoresistor-based pressure sensors, and flow sensors for the identification of airflow direction and velocity. The measurement of temperature was based on variations in linear resistance associated with changes in ambient temperature. The Au IDEs were covered with a water-absorbent polyimide layer to measure humidity, based on changes in the dielectric constant of the water-absorbent polyimide layer associated with changes in ambient moisture. To form the flow sensor and pressure sensor, eight pairs of heating and sensing resistors and four piezoresistors were manufactured on a membrane structure released after a back-etching process. The direction of airflow was determined according to fluctuations in the resistance of the sensors caused by air flowing through the sensor in a specific direction. The velocity of the airflow could then be obtained by summing the total variations measured by the sensing resistors. Finally, the electrical signals produced by temperature, humidity, pressure, airflow velocity and changes in direction were amplified and converted into voltage signals using an analogy circuit, connected between the MEMS-based sensors and the Octopus II sensor node [14]. Finally, the Octopus II sensor node converted the analog signals into digital signals and transmitted them wirelessly into a data logger or a computer, as shown in Figure 1.

## 2. Design

**Figure 2.**
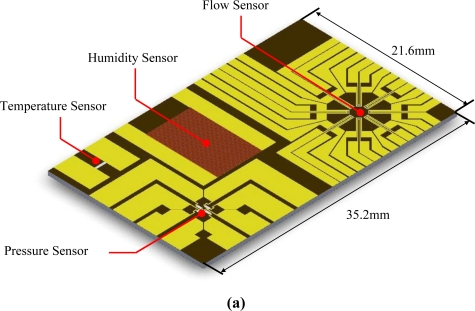

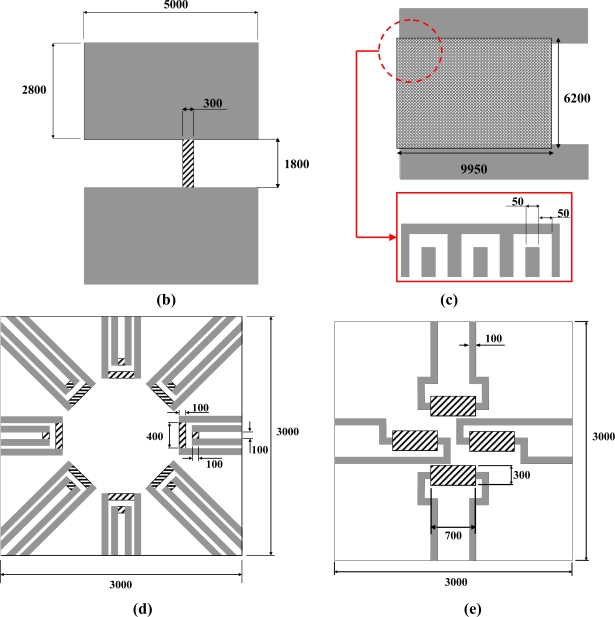
Configuration and dimensions of integrated sensor array of MEMS-based weather monitoring system: **(a)** overview, **(b)** RTD, **(c)** humidity sensor, **(d)** pressure sensor and **(e)** flow sensor. (unit: μm; 
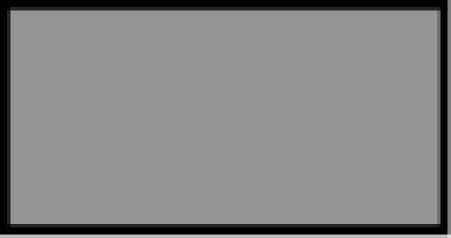
: Au; 
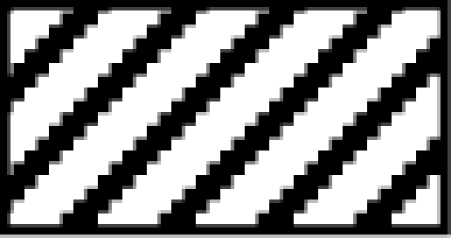
: Pt; 
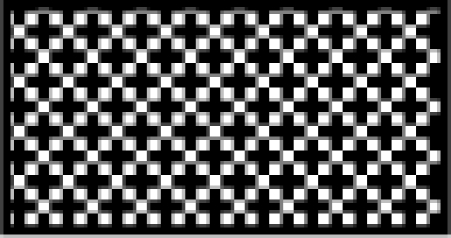
: Polyimide).

The sensor array of the proposed MEMS-based weather monitoring system was fabricated on a silicon nitride film over a silicon wafer utilizing platinum resistors as heating and sensing devices. Figure 2 illustrates the configuration and dimensions of the sensor array developed in the study. In Figure 2(a), the developed sensors comprise a Pt RTD, a humidity sensor with Au IDEs, a Pt-piezoresistor-based pressure sensor, and a flow sensor to determine the direction and velocity of airflow integrated onto a single chip. Note that Au was used for the bonding pads to connect the sensors to the circuits.

As shown in Figure 2(b), the Au bonding pads were deposited on the two edges of the Pt RTD. The size of the resistor used for the Pt RTD was 1,800 μm × 300 μm (length × width) Temperature measurements were based on linear variations in electrical resistance associated with changes in ambient temperature. Figure 2(c) shows the configuration of the humidity sensor, comprising a vapor absorbent film of polyimide (PW-1500, Toray Industries, Inc.) and coplanar Au IDEs. The piezoresistors used in the pressure sensor [Figure 2(c)] and the resistors utilized in the flow sensor [Figure 2(d)] were deposited over silicon nitride membranes of the same dimensions (3,000 μm × 3,000 μm), which were released by a back-etching process to form a heat insulating membrane over the thermal flow sensor. This was sealed with a back plate to obtain a vacuum cavity to house the pressure sensor. In Figure 2(e), the flow sensor is used to determine the direction and velocity of airflow using eight pairs of heating and sensing resistors on a membrane structure. The dimensions of the heaters and detectors are 100 μm × 100 μm and 400 μm × 100 μm, respectively.

As an electrical charge is applied to the heating resistors, the measurement of the direction of flow is based on the difference in relative output of the eight sensing resistors in response to variations in the temperature induced by the airflow due to the Joule effect. As the air passes over each pair of resistors in a particular direction, signal variations occur between pairs of resistors resulting in measurable changes in the resistance. Through manipulation and evaluation of the signals obtained from all of the sensing resistors, both the direction of flow and flow rate can be reliably determined.

In this study, a WSN platform device (Octopus II-A) [14] was used to perform analog/digital conversion and wireless transmission. This is an open-source visualization and control tool for sensor networks developed in the TinyOS environment (TinyOS is an open-source operating system designed for low-power wireless devices, such as sensor networks, ubiquitous computing, personal area networks, smart buildings and smart meters [15]). The MSP430F611 core processing chip in Octopus II was produced by Texas Instruments, Inc., and the Chipcon CC2420 chip was used for wireless communication according to the IEEE 802.15.4 specification.

## 3. Fabrication

**Figure 3.**
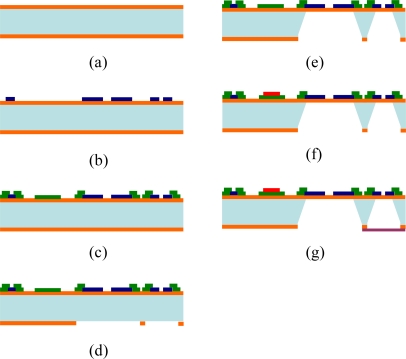
Simplified fabrication process of the sensor array of the MEMS-based weather monitoring system: **(a)** low-stress nitride deposition on silicon wafer, **(b)** electro-beam evaporation of Pt/Cr, **(c)** electro-beam evaporation of Au/Cr, **(d)** SF_6_ RIE, **(e)** back-etching, **(f)** spin-on polymide, **(g)** UV bonding and forming vacuum-sealed cavity at the pressure sensor.

In this study, Pt resistors were used as piezoresistors, heating and sensing resistors were used to measure deformations in the membrane and temperature, and Au was used as the bonding pads and lead wires connecting the external analog circuits. The low resistivity of Au reduced the resistance of the leads. Figure 3 shows the process of fabricating the sensor array of the MEMS-based weather monitoring system. Initially, a 1.0 μm low-stress nitride layer was deposited on both sides of a single-side polished silicon wafer. Before depositing the Pt, a thin layer of Cr (0.02 μm) was deposited as an adhesion layer. An electron beam evaporation process was then used to deposit a 0.1 μm layer of Pt. The same technique was used to add a layer of Au (0.4 μm) to serve as an electrode and to provide electrical leads [12]. To form the structure of the membrane, a back-etching nitride mask was patterned using an SF_6_ RIE plasma released in a KOH etchant (40 wt%, 85 °C, from J. T. Baker). A layer of polyimide (PW-1500, Toray Industries, Inc.) was then spun on and patterned as a moisture sensing layer (15 μm thick after curing) to play the role of humidity sensor. Finally, a layer of UV adhesive (OPAS-101, OPAS UV curing corp., Taiwan) was spun on a glass back plate and bonded with the chip of the sensor array in a negative pressure chamber to obtain a vacuum-sealed cavity for the following air pressure measurement.

## 4. Results and Discussion

**Figure 4.**
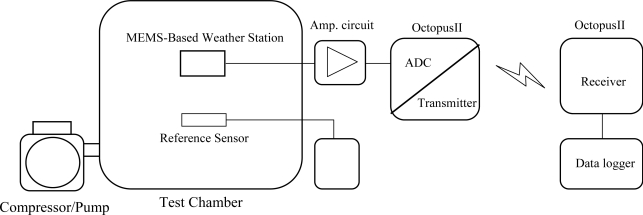
Schematic illustration of the wireless remote weather monitoring system for temperature, humidity and pressure.

This study conducted a systematic investigation of the performance of the fabricated sensor array of the MEMS-based weather monitoring system (Figure 4). The characteristic effects of temperature, humidity, and pressure sensors were carried out in a climate chamber (HRM-80FA, Terchy, Taiwan) setup in a vacuum chamber. The climate chamber was capable of providing a humidity range of 30% RH–95% RH and a temperature range of 20–85 °C. The temperature and humidity in the climate chamber could be adjusted separately and maintained at constant levels. For reference purposes, the humidity and temperature was also measured using a reference humidity/temperature meter (HT-3009, Lutron Electronics, Inc., Taiwan). Pressure measurements were carried out using a vacuum pump and an air compressor to vary the air pressure within the chamber. A reference pressure meter (PG-100, Nidec Copal Electronics Corp., Japan) was used to measure the actual pressure value to calibrate the response of the sensor. The flow sensor was tested in a wind tunnel in which the sensor was placed on a rotary table (LCPR60, TanLian E-O Co., Ltd., Taiwan). Airflow between 15 to 30 ms^−1^ was used to evaluate the signal response of the sensing resistors located at eight different points within the membrane. Note that for reference purposes the air flow rate was also measured using a reference anemometer (AM-4203, Lutron Electronics, Inc, Taiwan).

The electrical signals associated with temperature, humidity, pressure, airflow velocity and changes in direction were amplified and converted into signals of a specific voltage using an analog circuit, connected between the sensor array and the Octopus II sensor node. Through the Octopus II sensor node, the analog voltage signals were converted into digital signals and transmitted wirelessly to the data logger.

**Figure 5.**
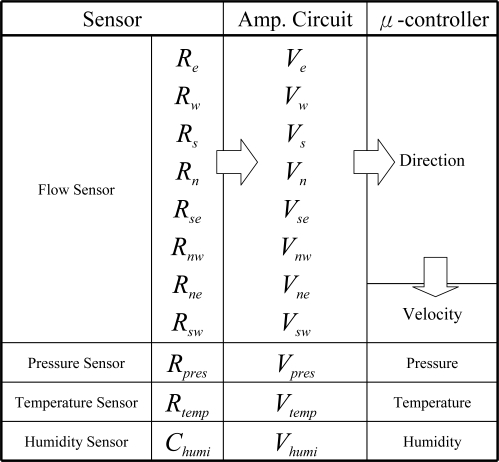
The signal determination path of the sensor array.

**Figure 6.**
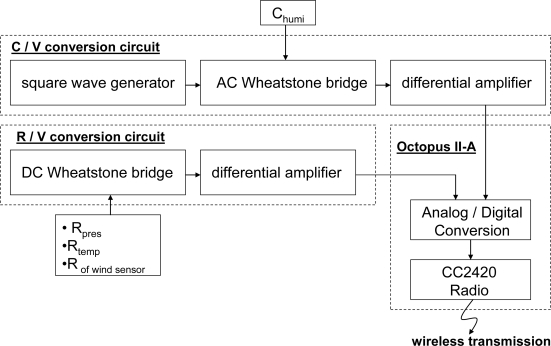
Schematic illustration of the analog circuit and signal conversion path of the sensor array in the MEMS-base weather sensor.

Figures 5 and 6 show the signal conversion and determination path of the signals from the sensor array. The resistance values of the eight flow sensors were obtained from the resistors located at eight points on the membrane, and the rate of airflow was obtained by evaluating the sum of the total variations between sensing resistors as the airflow passed over the flow sensor. The resistance signals could then be amplified and converted into voltage signals using Wheatstone bridge circuits. After the ADC operation of a μ-controller and the Octopus II-A, the digital signals were sent to the Octopus II-A receiver for the following data operation.

### 4.1. Temperature

**Figure 7.**
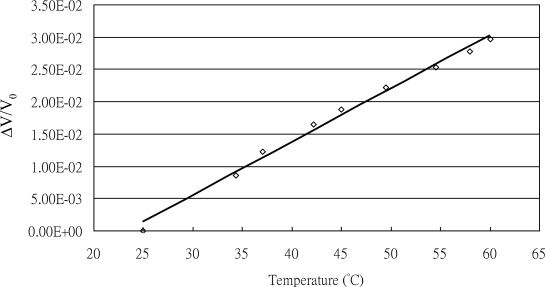
TCR test for the RTD (TCR = 8.2 × 10^−4^ °C^−1^).

The response of the RTD increased linearly with input temperature. The average TCR (temperature coefficient of resistance) value was 8.2 × 10^−4^ (°C^−1^) at a constant relative humidity of 60%. The relationship between the signal response and the ambient temperature is presented in Figure 7. The fitted equation of the data is given by:

(1) T=1,250 S+24.25

where *T* is the ambient temperature (°C) and *S* is the intensity (Δ*V/V_0_*). Note that a constant-current circuit was used for sensor operations and only a small current was allowed to pass through the resistor to prevent a self-heating effect.

### 4.2. Humidity

**Figure 8.**
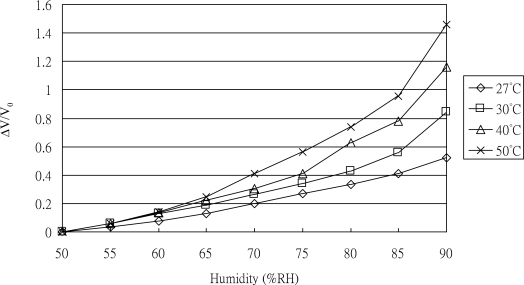
Experimental results of sensitivity to humidity ranging between 50 and 90% RH in steps of 5% RH at constant temperatures of 27, 30, 40, and 50 °C.

To determine the sensitivity of the sensor to humidity, the response was observed as humidity was increased from 50 to 90% RH in steps of 5% RH at constant temperatures of 27, 30, 40, and 50 °C, according to the reference humidity/temperature meter. The corresponding results are shown in Figure 8. It can be seen that the sensitivity to humidity was 16.9 pF/% RH at 27 °C, 21.4 pF/% RH at 30 °C, 27 pF/% RH at 40 °C, and 38.2 pF/% RH at 50 °C, respectively. Because of the vapor absorbed by the polymer film, the measured capacitance between the Au IDEs increased as the ambient humidity increased. The sensitivity to humidity also increased as the ambient temperature increased due to the temperature effect on the dielectric constant of the polyimide film.

### 4.3. Pressure

**Figure 9.**
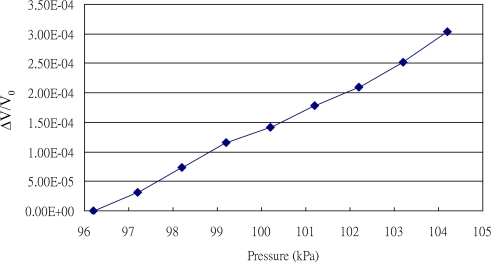
Experimental results of sensitivity to pressure ranging between 96 and 104 kPa.

As shown in Figure 9, the characteristics of the pressure sensor were investigated as the pressure was varied between 96 and 105 kPa. The measurement of pressure was carried out in a vacuum chamber connected to a vacuum pump and an air compressor to vary the pressure in the chamber. The experimental results indicated that the resistance of the pressure sensor increased linearly as the chamber pressure increased. The relationship between signal response and ambient pressure illustrated in the figure is given by:

(2) P=25,000  S+90

where *P* is the ambient pressure (°C) and *S* is the intensity (Δ*V/V_0_*).

### 4.4. Flow Rate and Direction

**Figure 10.**
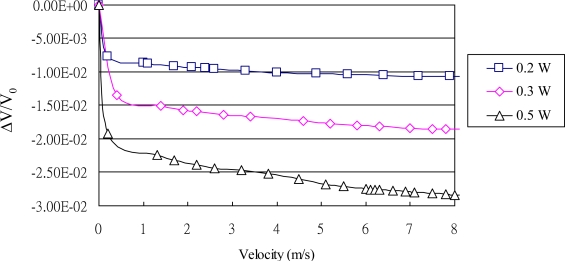
Experimental results of airflow rate ranging between 0 and 8 ms^−1^ with constant powers of 0.2, 0.3, and 0.5W.

In this study, the flow sensor comprised eight pairs of heating and sensing resistors located at eight points on a membrane. As an electrical charge was applied to the heating resistors, the direction of flow was measured according to differences in relative output of the eight sensing resistors as they responded to variations in temperature induced by the airflow due to the Joule effect. As the air passed over the pairs of the resistors in a particular direction, maximum variations in the signal could be measured between pairs of resistors, resulting in a measurable change in the resistance of the resistors. Through the evaluation and manipulation of the signals obtained from each sensing resistor, both the direction and rate of flow could be reliably determined. The rate of airflow was obtained by summing the variations in the signals from the sensing resistors (Figure 10) providing applied electrical values of 0.2, 0.3, and 0.5 W, representing airflow rates of 0 to 8 ms^−1^, respectively. It is clear that as the power consumption increased the sensitivity of the flow sensor increased due to the stronger heat transfer conditions caused by the higher power application.

**Figure 11.**
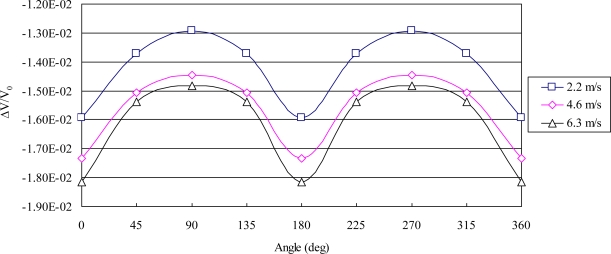
Experimental results of airflow direction with various rates of airflow.

The airflow direction was tested according to the airflow passing through the sensor. It was found that the greatest variation in resistance occurred between corresponding sensing resistors located in the same direction as the flow, with the smallest variation in response located in resistors aligned perpendicularly to the direction of airflow. The experimental results at flow rates of 2.2, 4.6, and 6.3 m/s are shown in Figure 11.

## 5. Conclusions

This study proposed a wireless remote weather monitoring system based on MEMS and WSN technology for monitoring temperature, humidity, pressure, flow rate and direction. The proposed sensor array used in the wireless remote weather monitoring system comprised a Pt RTD, a capacitive humidity sensor, a piezoresistive pressure sensor, and anemometers integrated through bulk-micromachining technology. The sensing signals were transmitted and received between the Octopus II-A sensor nodes using WSN Technology, after which the signal was amplified and the ADC of the original signals was processed. The experimental results show the response of the temperature sensor increases linearly with the ambient temperature. Its average TCR value was 8.2 × 10^−4^ (°C^−1^). The resistance of the pressure sensor increased linearly as the ambient air pressure increased with its average sensitivity calculated as 3.5 × 10^−2^ (Ω/kPa). The sensitivity of the humidity sensor increased with an increase in ambient temperature. due to the effect of temperature on its dielectric constant, which was determined to be 16.9, 21.4, 27.0, and 38.2 (pF/%RH) at 27 °C, 30 °C, 40 °C, and 50 °C, respectively. The rate of airflow was obtained by evaluating the sum of all variations between sensing resistors as the airflow passed over the flow sensor. The sensitivity to the rate of airflow was 4.2 × 10^−2^, 9.2 × 10^−2^, and 9.7 × 10^−2^ (Ω/ms^−1^) producing charges of 0.2, 0.3, and 0.5 W, respectively.
